# Classification of the Pathological Range of Motion in Low Back Pain Using Wearable Sensors and Machine Learning

**DOI:** 10.3390/s24030831

**Published:** 2024-01-27

**Authors:** Fernando Villalba-Meneses, Cesar Guevara, Alejandro B. Lojan, Mario G. Gualsaqui, Isaac Arias-Serrano, Paolo A. Velásquez-López, Diego Almeida-Galárraga, Andrés Tirado-Espín, Javier Marín, José J. Marín

**Affiliations:** 1 IDERGO (Research and Development in Ergonomics), I3A (Instituto de Investigación en Ingeniería de Aragón), University of Zaragoza, C/Mariano Esquillor s/n, 50018 Zaragoza, Spain; jmarinbone@unizar.es (J.M.); jjmarin@unizar.es (J.J.M.); 2 School of Biological Sciences and Engineering, Yachay Tech University, Hacienda San José s/n, San Miguel de Urcuquí 100119, Ecuador; alejandro.lojan@yachaytech.edu.ec (A.B.L.); mario.gualsaqui@yachaytech.edu.ec (M.G.G.); angel.arias@yachaytech.edu.ec (I.A.-S.); paolo.velasquez@yachaytech.edu.ec (P.A.V.-L.); dalmeida@yachaytech.edu.ec (D.A.-G.); 3 Department of Design and Manufacturing Engineering, University of Zaragoza, C/Mariano Esquillor s/n, 50018 Zaragoza, Spain; 4 Centro de Investigación en Mecatrónica y Sistemas Interactivos—MIST, Universidad Tecnológica Indoamérica, Quito 170103, Ecuador; cesarguevara@uti.edu.ec; 5 School of Mathematical and Computational Sciences, Yachay Tech University, Hacienda San José s/n, San Miguel de Urcuquí 100119, Ecuador; ctirado@yachaytech.edu.ec

**Keywords:** MoCap, classification, range of movement, machine learning, low back pain

## Abstract

Low back pain (LBP) is a highly common musculoskeletal condition and the leading cause of work absenteeism. This project aims to develop a medical test to help healthcare professionals decide on and assign physical treatment for patients with nonspecific LBP. The design uses machine learning (ML) models based on the classification of motion capture (MoCap) data obtained from the range of motion (ROM) exercises among healthy and clinically diagnosed patients with LBP from Imbabura–Ecuador. The following seven ML algorithms were tested for evaluation and comparison: logistic regression, decision tree, random forest, support vector machine (SVM), k-nearest neighbor (KNN), multilayer perceptron (MLP), and gradient boosting algorithms. All ML techniques obtained an accuracy above 80%, and three models (SVM, random forest, and MLP) obtained an accuracy of >90%. SVM was found to be the best-performing algorithm. This article aims to improve the applicability of inertial MoCap in healthcare by making use of precise spatiotemporal measurements with a data-driven treatment approach to improve the quality of life of people with chronic LBP.

## 1. Introduction

Low back pain (LBP), often referred to as lumbago, represents a group of symptoms and ailments concentrated in the lumbar region [1]. The significance of LBP is highlighted by its prevalent impact on health and daily functionality [2]; notably, the etiology of this condition is multidimensional. Experimental studies have indicated its origin in traumas to the spinal cord and adjacent structures. Another pivotal factor is age-related disk degeneration, amplifying the vulnerability of the spine to injury [3].

Statistically, LBP carries a substantial global health challenge. According to the 2015 global point prevalence rate of 7.3%, 540 million people were estimated to experience activity-limiting LBP at any given time. In 1990, the number of people with LBP at any given time was 377.5 million, which increased to 577.0 million in 2017 [4]. Currently, LBP is the leading cause of disability worldwide [5]. This widespread physical condition has notable economic consequences [6]. In 2016, a study estimated that USD $134.5 billion was spent on healthcare for LBP and neck pain in the United States [7]. By the early 2000s, the aggregated economic strain ascribed to LBP had soared to USD $26.3 billion [8].

However, discerning the true epidemiology of LBP requires an exhaustive understanding of its distribution and controlling factors, according to the Pan American Health Organization (PAHO) [9]. The existing literature related to LBP contains disparities and even contradictions [10], while some studies have correlated LBP with musculoskeletal injuries, age-related degeneration, spinal stenosis, and disk herniation [11,12], a broader spectrum of risk factors has emerged, including age, sex, geographical location, occupation, genetic predisposition, morphology, socioeconomic tier, and everyday practices [13]. Therefore, LBP diagnosis remains a multifaceted health challenge, marked by its widespread occurrence and substantial economic implications [4]. Recognizing the intricate interplay of its risk factors is vital for clinicians, public health strategists, and policymakers as they devise interventions to address this global ailment.

In contemporary clinical perspectives, LBP categorization is chiefly contingent on symptom duration. Acute LBP persists for less than 12 weeks, while the chronic variant endures beyond this period [14]. A noteworthy subset experiences recurrent episodes, and certain research introduces a sub-acute category characterized by a 6–12-week symptom persistence. The transition risk from the acute to the chronic phases is heightened for those suffering prolonged acute LBP episodes [15]. Notably, around 50–80% of the global populace is projected to face LBP challenges, with a substantial 90% likely to undergo intermittent episodes even post-initial alleviation [16]. The global burden of LBP has witnessed a significant surge, with the prevalence and years lived with disability (YLDs) escalating from 42.5 million (95% UI: 30.2 million–57.2 million) in 1990 to 64.9 million (95% UI: 46.5 million–87.4 million) in 2017, marking a 52.7% increase [4].

Diagnosing low back pain (LBP) traditionally prioritizes patient medical histories, symptomatology, co-morbidities, prior therapeutic approaches, and hematologic evaluations to rule out infections [17]. Complementing this, imaging techniques like X-rays and MRIs are regularly employed despite their often insufficient specificity for cases of nonspecific LBP [18]. These contemporary diagnostic methods can fall short of capturing the dynamic and functional impairments intrinsic to LBP, leading to a gap in comprehensive LBP management. In this context, the integration of motion capture (MoCap) technology, particularly when improved by inertial measurement units (IMUs) wearable sensors, signals a pivotal shift in this diagnostic landscape [19,20,21]. These compact devices, armed with triaxial accelerometers, gyroscopes, and magnetometers, provide a sophisticated, non-invasive means to detail the spatiotemporal and kinematic complexities of patient biomechanical movement, offering insights that static imaging cannot [19,21,22], while current approaches may rely on subjective assessments, MoCap introduces an objective and quantitative facet to the evaluation of LBP, enriching the diagnostic process with large dynamic data [23].

It should be noted that a considerable segment of LBP patients experience symptom reduction within the initial days without treatment [6], precluding the need for imaging, barring instances of tangible structural anomalies or infections. Then, physical examination remains a cornerstone, especially for assessing lower muscular structures and neurological function [11]. The need for innovation is underscored by the challenge of treating LBP without a precise diagnosis of its root causes [13]. Critically, MoCap’s true potential is realized when used in conjunction with traditional methods. This integration allows for a multidimensional diagnostic approach, where MoCap’s granular analysis of spinal movement complements the broader clinical picture obtained from patient history and imaging [17,18]. Such a synthesis of the registered raw data promises to refine our understanding of LBP, facilitating more tailored and efficacious treatments [21,23,24,25].

Furthermore, the evaluation of joint health frequently mandates the analysis of its range of motion (ROM) [26]. Historically, instruments such as goniometers and measurement tapes have dominated ROM assessments, exemplified by the Schober test’s ability to pinpoint mobility alterations [27]. Then, Washabaugh et al. underscored the repeatability and accuracy of inertial and optical sensors in appraising gait parameters, their assertions aligning with extant research regarding Lin’s concordance coefficient (LCC) and minimal detectable change (MDC) [19]. Zhao et al. highlighted MoCap’s ergonomics contributions, highlighting its preventive prowess against posture-induced afflictions in industrial arenas [20,28]. The recent trial for MoCap technology in LBP diagnosis lies in harnessing these data effectively, given its complexity and richness [23]. This paradigm shift, further propelled by the burgeoning relevance of artificial intelligence (AI) in medical contexts, hints at a future enriched by nuanced and anticipatory treatment modalities.

The intertwined nature of multidimensional data provided by MoCap demand robust analytic frameworks to process actionable data interpretations efficiently [29]. Among these innovations, machine learning (ML) methodologies aided by computational tools have emerged as invaluable allies in navigating these complexities [29,30,31]. These algorithms facilitate data categorization and expand horizons in medical applications [22,23,29]. However, the reliable efficacy of ML relies on testing and discerning the optimal algorithm from the myriad that are available, demanding substantial and specific samples of subjects accurately labeled for effective discriminative learning [29]. Simultaneously, inertial sensor-embedded wearables, capturing data longitudinally, elucidate the temporal dynamics of medical conditions like never before [22,32]. Raw data undergo rigorous algorithmic processing and classification, a proposition exemplified by Jourdan et al. [32]. The move toward data-driven, personalized interventions was also endorsed by Rabal-Pelay and their team [33], who leveraged inertial sensors for ROM assessments in industrial settings, thereby paving avenues for tailor-made remedial exercises.

The present study develops an ML-aided medical test to enhance health professionals’ decision-making process. This research harnesses IMU wearable sensor metrics to compare the ROM between the following two methodically labeled groups: healthy individuals and lumbago patients. Using these data, ML classification algorithms were trained to determine a categorical distinction between “normal” and “abnormal” ROM angles. The designated algorithms were rigorously tested using 10-fold cross-validation for the differentiation of pathological versus healthy states. Ultimately, the study offers detailed insights into lumbar vertebrae joint functionality, setting the stage for innovative therapeutic approaches and advanced patient care.

The focal aim is to harness inertial sensor metrics for comparing ROM in the following two cohorts: healthy individuals and lumbago patients. The ensuing data would be channeled into training classification algorithms, with the ultimate vision of generating reliable insights into lumbar vertebrae joint functionality. Achieving such granularity in diagnostics promises to reshape therapeutic avenues, fortifying patient care trajectories.

## 2. Materials and Methods

**Figure 1:**
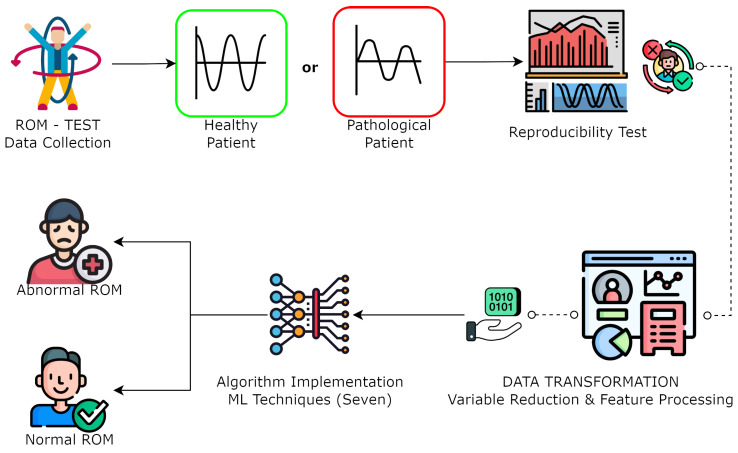
Methodology flow chart of the data acquisition and algorithm implementation for ROM classification. (Figure designed from Freepik illustrations).

In this section, we present a comprehensive figure that illustrates an overview of the experimental design employed throughout the methodology of this research study. Our study began with the collection of motion capture data from both pathological patients and healthy individuals. We conducted a reproducibility assessment to ensure data reliability and then rigorously selected significant kinematic variables through statistical analysis. In the subsequent data preprocessing step, we removed incomplete samples to create a consistent dataset. Standardization was applied to ensure uniformity among variables. Finally, we implemented machine learning algorithms for the classification of normal and abnormal ranges of motion, differentiating between healthy and pathological samples. The present methodology, as depicted in Figure 1, aimed to provide a robust means of characterizing pathological conditions through motion analysis.

### 2.1. Participants and Protocol

The inclusion criteria were individuals from Imbabura (Ecuador) aged from 18 to 65 years old, both male and female, divided into the following two groups: (1) pathological subjects with acute, subacute, and chronic LBP, and (2) a control group of subjects with no physical signs of pain. Exclusion criteria specified participants who had not received any form of physical or pharmacological treatment within the last six months to avoid bias. Over six months, 77 patients were evaluated through multiple repetitions of axial exercises using MoCap technology. Of those, 40 patients had been diagnosed with acute or chronic conditions, and the remaining 38 patients presented no signs of LBP.

### 2.2. Ethics Statement

Each patient signed a written informed consent form containing information about the procedure and data management. The protocol was developed according to the principles of the Declaration of Helsinki and approved by the bioethics committee of the Pontificia Universidad Católica del Ecuador N° EO-146-2022. All personal information from the subjects was kept anonymous in the present study.

### 2.3. Technology and Instrumentation

**Figure 2:**
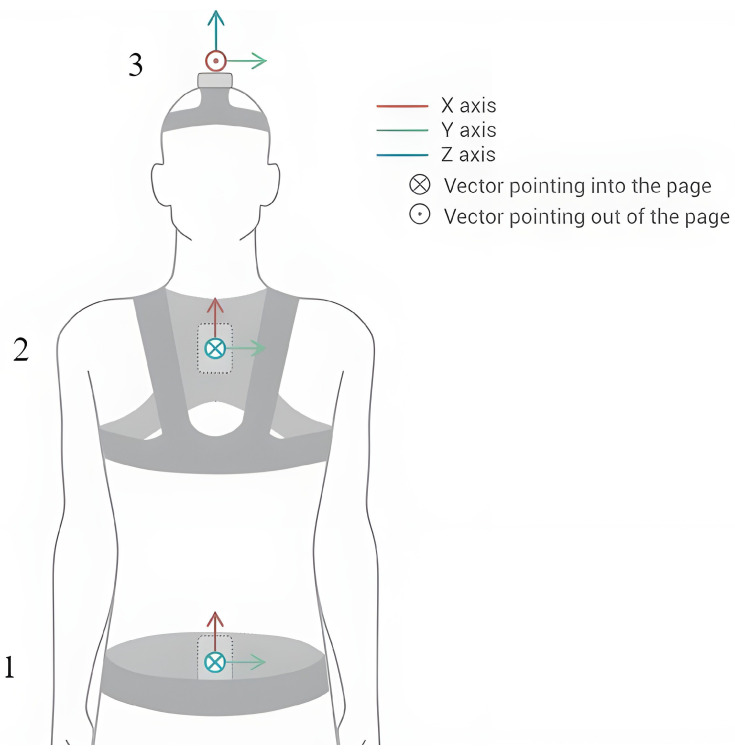
NGIMU orientation and placements on patient: Sensor 1 in the sacral region, sensor 2 in the cervical region, and sensor 3 is in the forehead. (Figure designed with Illustrator software (V26.0.1.731) by Adobe Inc., San Jose, CA, USA).

During the experimental phase, three next generation inertial motion unit sensors (NGIMU, x-io technologies, Bristol, UK) were used for motion data acquisition. Each NGIMU integrates a 3D gyroscope (Range: 2000°/s, Sample rate: 400 Hz, Resolution: 16-bit), a 3D accelerometer (Range: 16 g, Sample rate: 400 Hz, Resolution: 16-bit), and a 3D magnetometer (range: 1300 uT, Sample rate: 20 Hz, Resolution: 0.3 uT). The NGIMU utilizes the Madgwick algorithm to make an attitude and heading reference system (AHRS) with the outputs of the previously mentioned components. The NGIMU sensors were positioned as follows: one on the forehead, one on the seventh cervical vertebra (C7), and one in the sacrum region using elastic harnesses (see Figure 2). It was essential to fit the harnesses to the body of the patient tightly to prevent the sensor from registering unwanted movements. Spatiotemporal information is transmitted in the form of quaternions up to 400 Hz.

In order to evaluate the functional capacity of the lower region of the spinal column in relation to the pelvis using sensor units in a biomechanical assessment was performed with the following set of repetitive movements:

- Flexo-Extension (Flex): Starting in a standing up position, the patient leans forward with arms extended and reaches for the toes. The patient then moves back to a neutral standing position and then leans back in the sagittal plane (Rx);
- Rotation (Rot): With arms close to the chest cavity, the patient performs upper body rotations from left to right in the transversal plane (Ry), while keeping the waist fixed;
- Laterization (Lat): In a standing position and with a straighten back, the patient performs lateral movements on the frontal plane (Rz).

Patients repeated seven cycles of each exercise, maintaining uniform speed and constant performance and reaching the maximum ROM without discomfort. Trained personnel provided guidance in the correct execution of the exercises and operated the inertial sensor units. Patients were asked to perform an extra series of exercises if execution mistakes were made. Additional clinical information was taken relevant to the patients (age, sex, body mass index, educational level).

### 2.4. Data Preprocessing

The information acquired from the motion sensors was analyzed and preprocessed in real time using Move Human Sensors (MH) software (V19-07.011, University of Zaragoza, Zaragoza, Spain) developed by Marin et al. [34]. This software is versatile and can operate with any sensor system and a minimal number of sensors. For this study, the software was configured to employ three sensors, placed as described earlier. The software converts the quaternions from the sensors into rotation matrices and generates a report with various parameters such as angles, velocities, and relative positions. As the authors of the MH software explained, the software also calibrates a global coordinate system with the origin at the abdominal region at *t* = 0. We chose this software because it is suitable for analyzing lumbar movements, which is the main focus of our study; therefore, the fixed reference was designated in the sensor located in sacral position. The relative position of the other two sensors was obtained from this position. The sensor features was acquired along demographics parameters, are summarized in Table 1: the total length (total degrees traveled during the execution of the movement), the angular velocity (average speed of motion in degrees per second in the dorsal-lumbar movement), max range (overall range of motion in the dorsal-lumbar movement (maximum extension range plus maximum flexion range)), max/min value (average range of motion in the dorsal-lumbar movement (max is for flexion and min for extension)), and max/min speed (average speed in the dorsal-lumbar movement (max is for flexion and min for extension)).

The software MH provides an extensive detailed report with 48 variables for each exercise regarding angles, speed, and movement acceleration in tables, with reference values for consistency evaluation. Abnormal values are expected as a result of sensor interference due to inadequate exercise performance of the subjects, connectivity issues, or the presence of metallic objects. Filtration of unusable samples, such as incomplete series or missing values, is necessary to eliminate inconsistent samples. For individual missing datum, the values are estimated using the group mean, and samples with large portions of missing values can be eliminated to avoid bias. It is imperative to avoid the loss of clinical given the importance of number of samples for ML applications.

### 2.5. Feature Extraction

In this phase, we performed a series of examination and correction techniques and reduced the number of variables to avoid over-parametrization of the algorithms. First, we applied feature selection to homogenize the information available, such as working only with quantitative or qualitative data to reduce complexity. After the dataset extraction, we organized each sample for variable identification. It was necessary to convert the qualitative clinical data into quantitative information. We then performed feature selection to eliminate variables that did not contribute information relevant to the model, thus reducing the dimension of the samples.

The motion capture data and clinical variables were integrated into an optimized matrix, with 25 variables divided as follows: 21 in MoCap variables (seven metrics for each exercise: flexo/extension, rotation, and lateralization) and 4 in clinical variables (age, sex, body mass index, and educational level). An additional categorical variable was incorporated to predict the angle status (normal or abnormal) as the output in the ML models.

### 2.6. Database Validation

To assess the sensor data obtained from healthy and pathological subjects, we performed a Student’s *t*-test of independent samples to compare the characteristics extracted from the inertial sensors between the two groups of participants (n = 150). This test evaluates whether the means of two populations are equal or different, assuming that the samples come from normal distributions with equal or similar variances. We hypothesized that LBP would cause motor alterations that would reflect significant differences in the extracted characteristics. This test assumed that the means were equal, and we needed to reject this null hypothesis to support our hypothesis. We focused only on the data obtained from the inertial sensors because we wanted to support our hypothesis with objective and quantitative measures of motor performance. We did not perform any analysis of demographic variables, such as age and sex, because they are well-studied variables in the literature.

### 2.7. Model Implementation and Testing

The model presented aims to indicate whether the ROM angles correspond to a normal or abnormal state with classification algorithms to estimate an outcome in terms of a categorical variable. Hence, we evaluated the performance of the following seven ML algorithms that were employed: logistic regression, decision tree, random forest, SVM, k-nearest neighbor (KNN), multilayer perceptron (MLP), and gradient boosting algorithms to identify the best performance in classification of the collected samples of healthy and pathological LBP patients. We used the WEKA software (version 3.8.6, University of Waikato, Hamilton, New Zealand) for testing and training with a 10-fold cross-validation, WEKA, an open-source program developed by University of Waikato, New Zealand, based in the Java environment that does not require coding for ML applications. Hyperparameter tuning with trial and error was used to establish a range of significant changes for the acquired database (Table 2) to identify optimum parameter combination for each one of the algorithms tested; however, it is recommended to use a formal approach in the future.

Once the best hyperparameters were identified we used accuracy, precision, sensitivity, F-measure, and area under the curve for performance comparison. The error for each model was calculated using the root mean squared error (RMSE) and the mean absolute error (MAE).

## 3. Results

The final database included 150 samples selected for model testing. To ensure the accuracy and reliability of the training results, the selected samples were separated into the following two equal groups: 75 samples for pathological patients and 75 samples for healthy patients. For every sample, 25 independent variables were used to classify the dependent variable, of which the status of the patient was binary (pathological or healthy), see Supplementary Materials, Table S1.

### 3.1. Statistical Evaluation Results

Table 3 presents the results of the data validation with a Student’s *t*-test, showing the means, standard deviations (SD), and *t*, *p*, and *d* values for each parameter and group. A significance level of 0.05 was considered sufficient to reject the null hypothesis. Parameters that showed significant differences between groups were marked with an asterisk (*). To review other coefficients and raw statistical data, refer to the Supplementary Material.

As shown in Table 3, the *p*-values were <0.005, which indicates significant differences between the values of pathological and healthy groups. The feature values for the three axes of movement showed a longer mean length, a higher average velocity, a greater ROM, a larger value at maximum length and velocity, and a smaller value at minimum length and velocity for the healthy group, suggesting that the healthy group performed faster, more consistent, and more complex movements. By contrast, the pathological group presented a higher standard deviation in most characteristics compared to the healthy group, which implied that they performed more variable and less precise movements than the healthy group. The feature values were statistically significantly different and were useful for validating the results obtained by our ML model.

### 3.2. ML Results

Seven classification algorithms were trained and tested with 10-fold cross-validation to determine which models could more accurately identify and classify individual’s pathological and healthy status (Table 4). The results showed that all models had an accuracy rate of over 80%. Nevertheless, after performing parameter optimization, the SVM, random forest, and MLP algorithms were identified as the most effective models, with accuracy rates exceeding 90%. A similar finding was observed in the results for the sensitivity, precision, and F-measure. Moreover, random forest, logistic regression, SVM, and MLP had an area under the ROC curve (AURC) above 0.9. Regarding the error evaluation, we found that the KNN algorithm had the highest mean squared error (MSE) value of 0.3, indicating an inferior predictive capability compared with among the algorithms. The decision tree and gradient boosting algorithms exhibited poor performance in terms of the RMSE and Matthew’s correlation coefficient (MCC), with the lowest recorded values (Table 5).

**Figure 3:**
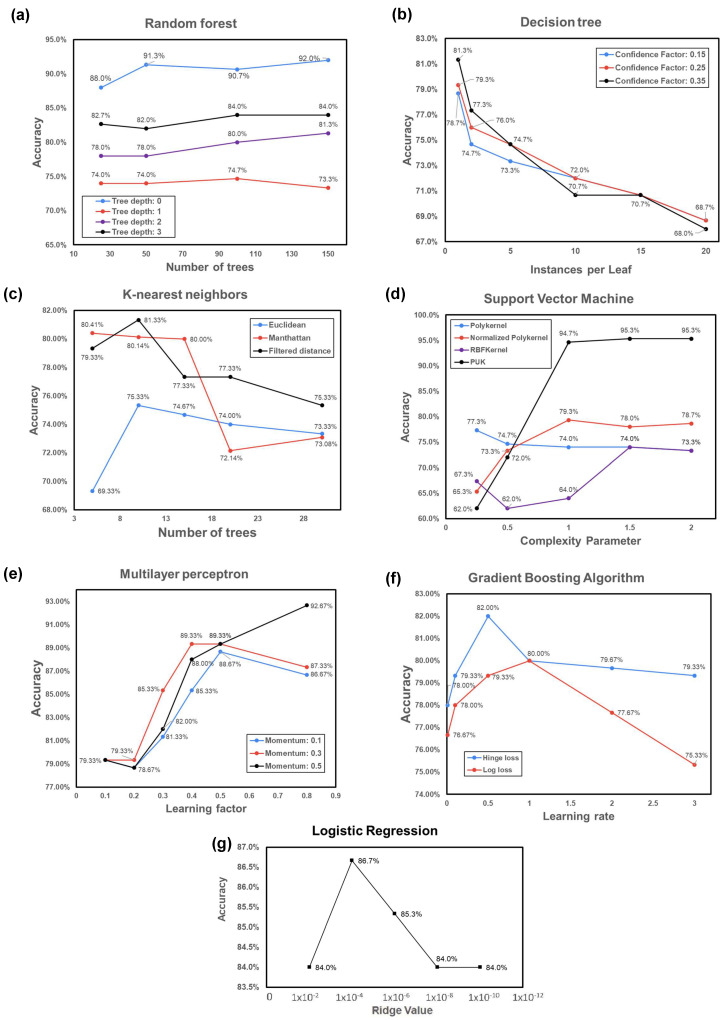
Performance plot of changes in hyperparameter optimization of the ML algorithms: (**a**) random forest; (**b**) decision tree; (**c**) KNN; (**d**) SVM; (**e**) MLP; (**f**) GBA; (**g**) logistic regression.

After optimizing the parameters, see Figure 3. The highest accuracy value corresponded to SVM, with a 95.3% accuracy based on a configuration of 1.5 in the complexity parameter and Pearson VII universal kernel (PUK). The second-best model was MLP, with an accuracy of 92.67% based on a configuration of 0.5 for momentum and a learning factor of 0.8. The random forest classification achieved 92% accuracy, with 150 trees and a tree depth equal to zero. The fourth most accurate model was the logistic regression algorithm, with an 86.7% accuracy by adjusting the ridge value to 1.0–4 and a batch size of 150.

The gradient boosting algorithm achieved an accuracy rate of 82% based on a learning rate of 0.5 and hinge loss to determine the optimal hyperplane. Finally, both the KNN and decision tree obtained an 81.3% accuracy using one instance per leaf with a confidence factor of 0.35 and 10 trees with filtered distances, respectively. The results demonstrated the effectiveness of these algorithms in accurately classifying the dataset.

## 4. Discussion

We developed the biomechanical analysis in this article to assist healthcare providers in making informed decisions regarding LBP treatment. The analysis involved categorizing clinical data from healthy and pathological individuals acquired with MoCap techniques. We evaluated the ability of seven ML classification algorithms to identify abnormal ranges of movement of subjects with LBP using motion capture data. After parameter optimization, the algorithms that showed the best performance, with accuracy rates of over 90%, were random forest, SVM, and MLP. These findings suggest that hyperparameter optimization plays a crucial role in enhancing the performance of the classification algorithms. The high accuracy rates achieved by these models indicate their potential for use in various applications, such as disease diagnosis and classification, anomaly detection, and predictive modeling. SVM was consistently the best-performing algorithm among the seven models tested, with the highest statistical results in all metrics and the lowest error for MAE and RMSE.

Inertial sensor units are beneficial because sensors can be placed in multiple locations on the body to perform a biomechanical assessment during multi-axial exercises. Having multiple sensors results in a significant amount of numerical information; however, it can lead to over-parametrization due to the high complexity of the data. A possible solution to increased parameter complexity in multidimensional time sequences produced by motion capture is to use ML supervised classifiers to accurately identify abnormalities in ROM angles and to provide a binary output, which offers a simpler understanding of lower back articular assessment. It was determined SVM algorithm was the most optimal for working with biomechanical information for LBP assessment.

Although the use of ML for motion capture data classification is a relatively new field of study, some investigations align with the present research study in data acquisition, ML incorporation, and results in terms of accuracy, see Table 6. Abdollahi, M. et al. focus their study on the classification of MoCap data from nonspecific low back pain (NLBP). However, their contribution lies in the classification of risk of developing NLBP (low, medium, high). Notably, they identify support vector machine (SVM) as the most accurate algorithm in their analysis [21]. Another notable article proposed by Bidabadi, S.S. et al. centers on classifying patients with lumbar radiculopathy, using MoCap gait data. The findings suggest that the combination of MoCap and ML can help discerning and characterization of lumbar pathologies. SVM and Naive Bayesian are among the best algorithms but the latter was not included in our study [22]. In addition, the article by Arshad, R. et al. evaluates chronic LBP with a trunk flexion-extension approach with MoCap for the classification of chronic LBP and non-LBP cases. This methodology shows feasibility, mirroring aspects of our study. However, our study diverges by offering a broader analysis with three different functional exercises (flex, tot, and lat.), expanding the scope beyond our singular exercise focus [35]. Our study builds upon the work of De la Torre et al., whose research involves a performance comparison of classification algorithms for MoCap triaxial movement exercises, while their emphasis is on cervical assessment, our study focuses specifically on the lumbar region. Both studies make significant contributions to the field, providing complementary insights into physical pathologies of the spine [36].

To obtain a robust and accurate classification of ROM values between the healthy and pathological groups, validation was conducted using the *t*-test as a discriminative tool. The Cohen’s d value indicated a significant distance between the means of both groups for each variable analyzed. These parameters provided information about the characteristics of the groups, as well as the dissemination of relevant variables for feature extraction. In our statistical analysis, we excluded age and gender as covariates. Our rationale for this choice was to examine the contrasts between healthy and pathological patients based on the sensor data. We implicit that these demographic factors had a substantial influence on the outcome variables. Furthermore, previous studies have already demonstrated significant differences in physiological parameters across age and gender groups. Arshad et al. [37] conducted a meta-analysis to examine the effect of age variation on the range of motion (ROM). Their findings suggested that the ROM was influenced by the age range of the participants in different studies. Hence, we applied the Student’s *t*-test to compare the mean values of the sensor variables between the two groups, as this was the most relevant statistical method for our study. The analysis revealed a statistically significant difference between the two groups. This finding is strongly supported by the existing literature. Sadler et al. [38] conducted a meta-analysis and found that lateral flexion was impaired in LBP patients. Amjad et al. [39] also showed the clinical relevance of the lumbar ROM in distinguishing healthy and LBP patients. A similar study with a comparable age range (18 to 65 years) to ours suggested that LBP could be predicted by the performance of different tasks and reported significant differences between healthy and chronic LBP patients [40]. We used a *t*-test to compare the means of the data obtained by sensors from the two groups of patients, healthy and pathological, and confirmed significant differences between them. To examine the effect of the sensor data on the ML models, a *t*-test was conducted without controlling for age and gender as covariates. In addition, we included these variables, along with other demographic and clinical information, as features in our machine learning models. These variables can enhance the accuracy and generalization of the predictions, as well as provide more insights about the characteristics of each group. This decision is supported by the literature of different cases of ML classification applied in biomedical field. For instance, a similar study used ML to analyze cervical pain with data from inertial sensor and considered age and sex as important inputs to avoid a bias in the classification [36].

This research project seeks to improve the practicality of inertial motion sensors in the context of healthcare, given their numerical precision in movement tracking, with the potential to enhance the quality of LBP treatment and diagnosis using a data-driven approach. Optimization in clinical and assistive settings through comprehensive data analysis and real-time monitoring can improve the accuracy and reliability of motion capture data for healthcare professionals, such as physical therapists and rehabilitation specialists.

The identification of abnormal ranges of movement through classification algorithms offers information about the diagnosis and localization of possible physical deviations that affect mobility in the lower back area across the different executed exercises. The applicability of this project is the development of a medical test to help healthcare professionals in the decision-making process and physical treatment assignment for patients with nonspecific LBP. With ROM, it is possible to further identify movement alterations in specific planes of movement (sagittal, transverse, and frontal) to set a baseline for clinician decision-making. In addition, it has the potential to help improve functional mobility with discerning changes before and after a physical treatment, therefore promoting personalized medicine to improve the overall quality of life for people with LBP. After model implementation, the classification algorithms showed promising results, given the elevated number of correctly classified instances reflected in the accuracy, precision, and recall of all models; however, since we are working with medical data, it is important to minimize incorrectly classified instances to avoid false positives and false negatives in diagnosis. Areas of improvement in this study could be related to the use of noncoding software alternatives, such as WEKA, which can restrict the process of hyperparameter tuning, although WEKA was selected based on the reproducibility of the methodology across the medical field aimed at accessibility for practitioners lacking coding expertise, exploring advanced hyperparameter tuning would indeed enhance our results that currently present favorable metrics despite software limitations and should be explored in the future. Further limitations of this work include the sample size of 150 individuals, meaning the variability of the data may not be fully captured. Although it would have been desirable to work with a larger sample size due to the COVID-19 pandemic and the implementation of strict biosecurity measures in healthcare centers at the moment of data collection, it was not possible to compile a larger sample. To solve this issue, multiple exercise repetitions per patient allowed duplication of the data samples from the same subject and increased algorithm training data. Despite this limitation, the results obtained are still significant and provide relevant information about the effectiveness of the different algorithms in classifying the dataset. A recommendation for future studies is to increase the number of participants to ensure a significantly larger dataset and acquire better classification results in terms of the described metrics and errors, as well as an in-depth analysis of hyperparameter tuning with coding alternatives. Another consideration would be to incorporate a pain/functionality questionnaire to explore additional variables of importance, such as the Roland–Morris questionnaire or the Oswestry LBP Disability Questionnaire, and to assess clinical viability. Finally, this study highlights that intelligent systems based on MoCap are viable not only for diagnosing LPB but also for assisting healthcare professionals in various diseases and across care phases.

## 5. Conclusions

The origin of LBP is uncommonly understood before treatment assignment; thus, new diagnostic tools are required to provide better insights into patient’s conditions and prevent the transition from acute to chronic cases. ROM assessment can help to estimate the physical state of the articulations of the body. By applying inertial sensor units, it is possible to acquire reliable metrics of the biomechanical capabilities of the spine by performing multi-axial range movements. Then, by incorporating sophisticated AI techniques, such as ML classifiers with motion sensors, we have demonstrated an effective method of identifying normal vs. abnormal ROM ranges, while all ML models showed an accuracy level > 80%, the most effective algorithms were found to be SVM with 95.3%, MLP with 93% and random forest with 92% in terms of accuracy. Both the software tools MoveHuman and WEKA can heavily simplify the application of ML for several applications, given that its noncoding interface can provide fast analysis for classification and regression models, and feature extraction is a great alternative for medical applications. The application of ML tools in coordination with motion capture for biomechanical ROM assessment can successfully estimate the physical state of the articulations of the lumbar spine region. By combining data analysis using ML techniques and current diagnostic protocols, it is possible to obtain more efficient results that help in the diagnosis of patients with LBP affecting range mobility.

## Figures and Tables

**Table 1:** Feature description from clinical data acquisition.

Class	Type	Variables	Movement Type
		Total Length (°)	
		Angular Velocity (°/s)	Flexo-Extension
Normal/Abnormal	Motion Capture Data	Max Range (°)	Rotation
ROM		Max/Min Value (°)	Laterization
		Max/Min Speed (°/s)	
	Demographics	Age, Sex, Body mass index (BMI), Educational level.	

**Table 2:** Hyperparameter adjustment for each classification model.

Classificator Model	Parameter Adjustment
Logistic Regression	Ridge Value in five segments from 1.0 $\times \;10^{-2}$ to 1.0 $\times \;10^{-10}$.
Decision tree	Confidence factor (0.15, 0.25, 0.35) and Instances per leaf (1, 2, 5, 10, 15, 20).
Random Forest	Tree depth (0, 1, 2, 3) and Number of trees (25, 50, 100, 150).
SVM	Complexity parameter (0.25, 0.5, 1, 1.5, 2). Tolerance 10 $\times \;10^{-3}$.
	Kernel variation (Polykernel, Normalized Polykernel, RBF, PUK).
K-nearest neighbors	Distance metric: Euclidean, Manhattan and Filtered distance.
	Number of trees (5, 15, 15, 20, 30).
Multilayer perceptron	Momentum (0.1, 0.3, 0.5). Learning factor: in six segments from 0.1 to 0.8.
Gradient boosting	Learning rate: 0.01, 0.1, 0.5, 1, 2, 3. Loss function: hinge loss and Log loss.

**Table 3:** *t*-test results of MoCap variables comparing healthy and pathological groups.

	Healthy (n = 75)	Pathologic (n = 75)	*t*	*p*	*d*
	$\bar{x}$ (SD)	$\bar{x}$ (SD)			
Flexion-Extension (Frontal axis) total length (°) *	104.61 (25.15)	87.21 (24.98)	4.250	<0.001	0.694
Flexion-Extension (Frontal axis) angular velocity (°/s) *	42.02 (11.84)	35.76 (14.57)	2.888	0.004	0.472
Flexion-Extension (Frontal axis) max range (°) *	83.99 (16.28)	71.60 (17.85)	4.442	<0.001	0.725
Flexion-Extension (Frontal axis) max value (°) *	62.13 (10.75)	55.53 (14.24)	3.204	0.002	0.523
Flexion-Extension (Frontal axis) min value (°) *	−22.56 (11.03)	−16.07 (7.95)	−0.137	<0.001	−0.676
Flexion-Extension (Frontal axis) max speed (°/s) *	117.46 (33.32)	91.59 (37.12)	4.492	<0.001	0.734
Flexion-Extension (Frontal axis) min speed (°/s) *	−92.21 (28.92)	−84.02 (30.99)	−1.674	0.096	−0.273
Rotation (Longitudinal axis) max length (°) *	98.82 (18.86)	87.484 (23.60)	3.250	0.001	0.531
Rotation (Longitudinal axis) angular velocity (°/s) *	45.15 (12.12)	38.10 (15.10)	3.153	0.002	0.515
Rotation (Longitudinal axis) max range (°) *	78.72 (12.50)	69.40 (15.51)	4.052	<0.001	0.662
Rotation (Longitudinal axis) max value (°) *	38.51 (6.76)	34.40 (8.49)	3.283	0.001	0.536
Rotation (Longitudinal axis) min value (°) *	−40.21 (6.71)	−35.0 (7.70)	−4.416	<0.001	−0.721
Rotation (Longitudinal axis) max speed (°/s) *	96.72 (25.15)	83.38 (30.63)	2.915	0.004	0.476
Rotation (Longitudinal axis) min speed (°/s) *	−95.93 (23.44)	−81.61 (29.61)	−3.284	0.001	−0.536
Laterization (Sagittal axis) total length (°) *	98.69 (17.49)	77.97 (23.93)	6.053	<0.001	0.988
Laterization (Sagittal axis) angular velocity (°/s) *	42.10 (9.89)	33.67 (13.69)	4.326	<0.001	0.706
Laterization (Sagittal axis) max range (°) *	77.09 (12.49)	61.78 (16.02)	6.527	<0.001	1.066
Laterization (Sagittal axis) max value (°) *	40.07 (6.77)	31.83 (8.16)	6.727	<0.001	1.099
Laterization (Sagittal axis) min value (°) *	−37.02 (6.52)	−29.95 (8.45)	−5.738	<0.001	−0.937
Laterization (Sagittal axis) max speed (°/s) *	79.77 (19.86)	64.02 (25.72)	4.197	<0.001	0.685
Laterization (Sagittal axis) min speed (°/s) *	−80.74 (20.16)	−65.31 (24.48)	−4.213	<0.001	−0.688

$\bar{x}$ = mean, SD = Standard deviation, *t* = *t*-value, *p* = bilateral significance, *d* = Cohen’s d. Parameters that showed significant differences between groups were marked with an asterisk (*).

**Table 4:** Classification ML algorithms metric results.

Classifier Type	Accuracy (%)	Precision	Sensitivity	F-Measure	AURC
Logistic Regression	86.67%	0.867	0.867	0.867	0.908
Decision tree	81.33%	0.814	0.813	0.813	0.859
Random Forest	92%	0.92	0.92	0.92	0.977
SVM	95.33%	0.957	0.953	0.953	0.953
K-nearest neighbors	81.33%	0.813	0.813	0.813	0.869
Multilayer perceptron	93%	0.927	0.927	0.927	0.936
Gradient boosting	82.00%	0.82	0.82	0.82	0.82

**Table 5:** Error evaluation of ML results.

Classifier Type	Correct Class	Incorrect Class	MAE	RMSE	MCC
Logistic Regression	130	20	0.1523	0.3283	0.734
Decision tree	122	28	0.1937	0.4157	0.627
Random Forest	138	12	0.2269	0.2844	0.84
SVM	143	7	0.0467	0.216	0.911
K-nearest neighbors	122	28	0.3107	0.3958	0.627
Multilayer perceptron	139	11	0.1277	0.2755	0.854
Gradient boosting	123	27	0.18	0.4243	0.64

**Table 6:** Related works on ML classification in biomechanical applications.

Aim of the Study	Algorithm Type	Accuracy	Ref.
Lumbar radiculopathy assessment using gait biomechanics	Random Forest	88.40%	[22]
	Support Vector Machine	86.90%	
	Naive Bayes	86.10%	
Cervical Pain Assessment	Logistic Regression	86.60%	[36]
	Support Vector Machine	95.40%	
	Decision tree	83.70%	
	Random Forest	87.70%	
	Neural network algorithm	91.80%	
	K-Nearest Neighbors	83.40%	
	Gradient boosting	87.10%	
Categorize Nonspecific Low Back Pain	Support Vector Machine	∼75%	[21]
	Multi-layer perceptron (MLP)	60%	
Chronic Low Back Pain Identification	Brute-Force K-Nearest Neighbors	63.00%	[35]
	Support Vector Machine	72.00%	
	Radial basis function	52.00%	
	Decision Tree	66.00%	
	Random Forest	78.00%	
	Adaptive boosting	68.00%	
	Gaussian Naive Bayes	79.00%	

## Data Availability

The original contributions presented in the study are included in the article/supplementary material, further inquiries can be directed to the corresponding author.

## Supplementary Materials

The following supporting information can be downloaded at: https://www.mdpi.com/article/10.3390/s24030831/s1. This article contains spreadsheet of the results of data collection and complete statistics tables from both groups, Table S1: LBP_Dataset, Table S2: Raw statistics of the Healthy and Pathological Group, Table S3: Independent Sample Test from both groups, Table S4: Independent Samples Effect Sizes.
