# Supplementary material for: Classification of the Pathological Range of Motion in Low Back Pain Using Wearable Sensors and Machine Learning
Source: Sensors (Basel). 2024 Jan 27;24(3):831. doi: 10.3390/s24030831 (PMC10857033; doi:10.3390/s24030831)
Supplement: Supplementary file 1 [file sensors-24-00831-s001.zip › Tables S2-S4.pdf]

Table S2. Raw statistics of the Healthy and Pathological Group.

Group Statistics					
	**State**	**N**	**Mean**	**Std. Deviation**	**Std. Error Mean**
Flexion-Extension (Frontal axis) total length (°)	Healthy	75	104.6099071409	25.15439792946	2.90457968317
	Pathological	75	87.2123917451	24.98525195654	2.88504838858
Flexion-Extension (Frontal axis) angular velocity (°/s)	Healthy	75	42.01754850973	11.838397370221	1.366980381694
	Pathological	75	35.75737557088	14.566584623693	1.682004310733
Flexion-Extension (Frontal axis) max range (°)	Healthy	75	83.9940422404	16.27895200765	1.87973146475
	Pathological	75	71.6035092065	17.85171657143	2.06133867360
Flexion-Extension (Frontal axis) max value (°)	Healthy	75	62.1324809656	10.75174521944	1.24150459934
	Pathological	75	55.5320776380	14.23631007345	1.64386749063
Flexion-Extension (Frontal axis) min value (°)	Healthy	75	-22.56532880041	11.029395245058	1.273564862747
	Pathological	75	-16.07143156759	7.947855977797	.917739357652
Flexion-Extension (Frontal axis) max speed (°/s)	Healthy	75	117.4639676831	33.31579127239	3.84697621188
	Pathological	75	91.5928370787	37.11871218411	4.28609969429
Flexion-Extension (Frontal axis) min speed (°/s)	Healthy	75	-92.2110028783	28.91931819524	3.33931522896
	Pathological	75	-84.0193387055	30.98899760357	3.57830122167
Flexion-Extension (Frontal axis) Speed Harmony (°/s)	Healthy	75	-.55034578191	.134103919083	.015484986756
	Pathological	75	-.59204414396	.143123430425	.016526470217
Rotation (Longitudinal axis) total length (°)	Healthy	75	98.8218647033	18.86494160786	2.17833582311

	Pathological	75	87.4840263227	23.59679801473	2.72472353716
Rotation (Longitudinal axis) angular velocity (°/s)	Healthy	75	45.1473483475	12.12509520909	1.40008539658
	Pathological	75	38.0959401820	15.10079538200	1.74368965575
Rotation (Longitudinal axis) max range (°)	Healthy	75	78.7188782581	12.50422359195	1.44386337136
	Pathological	75	69.3969534108	15.51400726869	1.79140325456
Rotation (Longitudinal axis) max value (°)	Healthy	75	38.5115326231	6.76018816307	.78059929114
	Pathological	75	34.3978137489	8.48850029151	.98016758566
Rotation (Longitudinal axis) min value (°)	Healthy	75	-40.2073456361	6.70814814281	.77459022720
	Pathological	75	-34.9991396620	7.70283269705	.88944650623
Rotation (Longitudinal axis) max speed (°/s)	Healthy	75	96.7213783133	25.14777087815	2.90381445720
	Pathological	75	83.3823404319	30.63426996251	3.53734080186
Rotation (Longitudinal axis) min speed (°/s)	Healthy	75	-95.9354483597	23.44477021921	2.70716887943
	Pathological	75	-81.6118858641	29.60993768489	3.41906109861
Rotation (Longitudinal axis) speed harmony (°/s)	Healthy	75	-.59877726432	.138512142401	.015994004540
	Pathological	75	-.57702491489	.139813361347	.016144256362
Laterization (Sagittal axis) Total Length (°)	Healthy	75	98.6878282457	17.49016805190	2.01959064659
	Pathological	75	77.9673070824	23.93475987780	2.76374801169
Laterization (Sagittal axis) angular velocity (°/s)	Healthy	75	42.10494142600	9.894845439146	1.142558335576
	Pathological	75	33.66563088285	13.692978735897	1.581128991836
Laterization (Sagittal axis) max range (°)	Healthy	75	77.0943477249	12.49068361324	1.44229990929
	Pathological	75	61.7832643621	16.02268927451	1.85014079316

Laterization (Sagittal axis) max value (°)	Healthy	75	40.0710514899	6.77079003664	.78182349006
	Pathological	75	31.8352556375	8.15858808119	.94207260498
Laterization (Sagittal axis) min value (°)	Healthy	75	-37.02329623387	6.524195485881	.753349204004
	Pathological	75	-29.94800872479	8.453715272320	.976150957625
Laterization (Sagittal axis) max speed (°/s)	Healthy	75	79.7717408824	19.85868273678	2.29308316477
	Pathological	75	64.0221178512	25.72353547178	2.97029802583
Laterization (Sagittal axis) min speed (°/s)	Healthy	75	-80.7410002301	20.16459806429	2.32840722410
	Pathological	75	-65.3123756316	24.47786501594	2.82646039123
Laterization (Sagittal axis) speed harmony (°/s)	Healthy	75	-.65644624315	.151417357877	.017484170466
	Pathological	75	-.67780276156	.143678263342	.016590536803

Table S3. Independent Sample Test from both groups.

Independent Samples Test										
		Levene's Test for Equality of Variances		t-test for Equality of Means						
									95% Confidence Interval of the Difference	
		F	Sig.	t	df	Sig. (2-tailed)	Mean Difference	Std. Error Difference	Lower	Upper
Flexion-Extension (Frontal axis) total length (°)	Equal variances assumed	.044	.834	4.250	148	.000	17.39751539587	4.09390856521	9.30745059335	25.48758019838
	Equal variances not assumed			4.250	147.993	.000	17.39751539587	4.09390856521	9.30744755777	25.48758323397
Flexion-Extension (Frontal axis) angular velocity (°/s)	Equal variances assumed	2.130	.147	2.888	148	.004	6.260172938853	2.167434858366	1.977056161795	10.543289715912
	Equal variances not assumed			2.888	142.061	.004	6.260172938853	2.167434858366	1.975579779290	10.544766098417
Flexion-Extension (Frontal axis) max range (°)	Equal variances assumed	.181	.671	4.442	148	.000	12.39053303387	2.78971459236	6.87771527713	17.90335079060
	Equal variances not assumed			4.442	146.759	.000	12.39053303387	2.78971459236	6.87733091734	17.90373515040
Flexion-Extension (Frontal axis) max value (°)	Equal variances assumed	3.926	.049	3.204	148	.002	6.60040332760	2.06000825167	2.52957468445	10.67123197075
	Equal variances not assumed			3.204	137.694	.002	6.60040332760	2.06000825167	2.52706171194	10.67374494326

Flexion-Extension (Frontal axis) min value (°)	Equal variances assumed	9.142	.003	-4.137	148	.000	-6.493897232827	1.569781191188	-9.595977154784	-3.391817310869
	Equal variances not assumed			-4.137	134.531	.000	-6.493897232827	1.569781191188	-9.598539311758	-3.389255153896
Flexion-Extension (Frontal axis) max speed (°/s)	Equal variances assumed	2.160	.144	4.492	148	.000	25.87113060440	5.75932952384	14.48998998421	37.25227122459
	Equal variances not assumed			4.492	146.304	.000	25.87113060440	5.75932952384	14.48890230471	37.25335890409
Flexion-Extension (Frontal axis) min speed (°/s)	Equal variances assumed	2.258	.135	-1.674	148	.096	-8.19166417280	4.89441169410	-17.86362121452	1.48029286892
	Equal variances not assumed			-1.674	147.298	.096	-8.19166417280	4.89441169410	-17.86400093691	1.48067259131
Flexion-Extension (Frontal axis) Speed Harmony (°/s)	Equal variances assumed	.332	.566	1.841	148	.068	.041698362053	.022647495064	-.003055862366	.086452586473
	Equal variances not assumed			1.841	147.377	.068	.041698362053	.022647495064	-.003057421068	.086454145175
Rotation (Longitudinal axis) total length (°)	Equal variances assumed	3.872	.051	3.250	148	.001	11.33783838067	3.48844740712	4.44423899168	18.23143776965
	Equal variances not assumed			3.250	141.159	.001	11.33783838067	3.48844740712	4.44148411352	18.23419264781

Rotation (Longitudinal axis) angular velocity (°/s)	Equal variances assumed	4.658	.033	3.153	148	.002	7.05140816547	2.23622287201	2.63235784735	11.47045848358
	Equal variances not assumed			3.153	141.402	.002	7.05140816547	2.23622287201	2.63065752515	11.47215880579
Rotation (Longitudinal axis) max range (°)	Equal variances assumed	1.777	.185	4.052	148	.000	9.32192484733	2.30084051068	4.77518215995	13.86866753471
	Equal variances not assumed			4.052	141.612	.000	9.32192484733	2.30084051068	4.77349085873	13.87035883593
Rotation (Longitudinal axis) max value (°)	Equal variances assumed	4.943	.028	3.283	148	.001	4.11371887413	1.25302184710	1.63759421708	6.58984353119
	Equal variances not assumed			3.283	140.940	.001	4.11371887413	1.25302184710	1.63657144383	6.59086630443
Rotation (Longitudinal axis) min value (°)	Equal variances assumed	.124	.726	-4.416	148	.000	-5.20820597413	1.17945118913	-7.53894599908	-2.87746594918
	Equal variances not assumed			-4.416	145.258	.000	-5.20820597413	1.17945118913	-7.53930870499	-2.87710324328
Rotation (Longitudinal axis) max speed (°/s)	Equal variances assumed	3.794	.053	2.915	148	.004	13.33903788147	4.57656184819	4.29519106984	22.38288469309
	Equal variances not assumed			2.915	142.588	.004	13.33903788147	4.57656184819	4.29236055848	22.38571520446
Rotation (Longitudinal axis) min speed (°/s)	Equal variances assumed	5.912	.016	-3.284	148	.001	-14.32356249560	4.36104828428	-22.94152810589	-5.70559688531

	Equal variances not assumed			-3.284	140.606	.001	-14.32356249560	4.36104828428	-22.94526507584	-5.70185991536
Rotation (Longitudinal axis) speed harmony (°/s)	Equal variances assumed	.142	.707	-.957	148	.340	-.021752349427	.022725430573	-.066660583959	.023155885105
	Equal variances not assumed			-.957	147.987	.340	-.021752349427	.022725430573	-.066660616321	.023155917468
Laterization (Sagittal axis) Total Length (°)	Equal variances assumed	4.513	.035	6.053	148	.000	20.72052116333	3.42301759445	13.95621909891	27.48482322775
	Equal variances not assumed			6.053	135.495	.000	20.72052116333	3.42301759445	13.95106941443	27.48997291224
Laterization (Sagittal axis) angular velocity (°/s)	Equal variances assumed	3.225	.075	4.326	148	.000	8.439310543147	1.950745611047	4.584398261840	12.294222824453
	Equal variances not assumed			4.326	134.725	.000	8.439310543147	1.950745611047	4.581264761297	12.297356324997
Laterization (Sagittal axis) max range (°)	Equal variances assumed	1.471	.227	6.527	148	.000	15.31108336280	2.34590067625	10.67529626867	19.94687045693
	Equal variances not assumed			6.527	139.684	.000	15.31108336280	2.34590067625	10.67302017506	19.94914655054
Laterization (Sagittal axis) max value (°)	Equal variances assumed	.668	.415	6.727	148	.000	8.23579585240	1.22423394931	5.81655960772	10.65503209708
	Equal variances not assumed			6.727	143.137	.000	8.23579585240	1.22423394931	5.81588191585	10.65570978895

Laterization (Sagittal axis) min value (°)	Equal variances assumed	1.884	.172	-5.738	148	.000	-7.075287509080	1.233047328875	-9.511940071360	-4.638634946800
	Equal variances not assumed			-5.738	139.067	.000	-7.075287509080	1.233047328875	-9.513230866025	-4.637344152135
Laterization (Sagittal axis) max speed (°/s)	Equal variances assumed	2.423	.122	4.197	148	.000	15.74962303120	3.75245263298	8.33431697695	23.16492908545
	Equal variances not assumed			4.197	139.087	.000	15.74962303120	3.75245263298	8.33039816527	23.16884789713
Laterization (Sagittal axis) min speed (°/s)	Equal variances assumed	.865	.354	-4.213	148	.000	-15.42862459853	3.66201563957	-22.66521607585	-8.19203312122
	Equal variances not assumed			-4.213	142.767	.000	-15.42862459853	3.66201563957	-22.66740306843	-8.18984612864
Laterization (Sagittal axis) speed harmony (°/s)	Equal variances assumed	1.300	.256	.886	148	.377	.021356518413	.024102741095	-.026273450423	.068986487250
	Equal variances not assumed			.886	147.595	.377	.021356518413	.024102741095	-.026274529089	.068987565915

Table S4. Independent Samples Effect Sizes

Independent Samples Effect Sizes					
				95% Confidence Interval	
		Standardizer[a]	Point Estimate	Lower	Upper
Flexion-Extension (Frontal axis) total length (°)	Cohen's d	25.06996759596	.694	.363	1.023
	Hedges' correction	25.19791190762	.690	.361	1.017
	Glass's delta	24.98525195654	.696	.355	1.033
Flexion-Extension (Frontal axis) angular velocity (°/s)	Cohen's d	13.272773634295	.472	.146	.795
	Hedges' correction	13.340511092666	.469	.146	.791
	Glass's delta	14.566584623693	.430	.101	.756
Flexion-Extension (Frontal axis) max range (°)	Cohen's d	17.08344319823	.725	.394	1.055
	Hedges' correction	17.17062836798	.722	.392	1.049
	Glass's delta	17.85171657143	.694	.353	1.031
Flexion-Extension (Frontal axis) max value (°)	Cohen's d	12.61492270629	.523	.197	.848
	Hedges' correction	12.67930282948	.521	.196	.844
	Glass's delta	14.23631007345	.464	.134	.791
Flexion-Extension (Frontal axis) min value (°)	Cohen's d	9.612907315571	-.676	-1.004	-.345
	Hedges' correction	9.661966685305	-.672	-.999	-.344
	Glass's delta	7.947855977797	-.817	-1.161	-.469
Flexion-Extension (Frontal axis) max speed (°/s)	Cohen's d	35.26854648488	.734	.402	1.063

	Hedges' correction	35.44853913488	.730	.400	1.058
	Glass's delta	37.11871218411	.697	.356	1.034
Flexion-Extension (Frontal axis) min speed (°/s)	Cohen's d	29.97202810414	-.273	-.594	.049
	Hedges' correction	30.12499002921	-.272	-.591	.048
	Glass's delta	30.98899760357	-.264	-.586	.059
Flexion-Extension (Frontal axis) Speed Harmony (°/s)	Cohen's d	.138687017147	.301	-.022	.622
	Hedges' correction	.139394804856	.299	-.022	.619
	Glass's delta	.143123430425	.291	-.033	.614
Rotation (Longitudinal axis) total length (°)	Cohen's d	21.36229035492	.531	.204	.856
	Hedges' correction	21.47131257541	.528	.203	.851
	Glass's delta	23.59679801473	.480	.150	.808
Rotation (Longitudinal axis) angular velocity (°/s)	Cohen's d	13.69401246893	.515	.189	.839
	Hedges' correction	13.76389971519	.512	.188	.835
	Glass's delta	15.10079538200	.467	.137	.794
Rotation (Longitudinal axis) max range (°)	Cohen's d	14.08971307675	.662	.332	.989
	Hedges' correction	14.16161977683	.658	.330	.984
	Glass's delta	15.51400726869	.601	.265	.933
Rotation (Longitudinal axis) max value (°)	Cohen's d	7.67316040491	.536	.209	.861
	Hedges' correction	7.71232029702	.533	.208	.857
	Glass's delta	8.48850029151	.485	.154	.812
Rotation (Longitudinal axis) min value (°)	Cohen's d	7.22263397469	-.721	-1.050	-.390
	Hedges' correction	7.25949460998	-.717	-1.045	-.388

	Glass's delta	7.70283269705	-.676	-1.012	-.336
Rotation (Longitudinal axis) max speed (°/s)	Cohen's d	28.02560326091	.476	.151	.800
	Hedges' correction	28.16863162759	.474	.150	.796
	Glass's delta	30.63426996251	.435	.106	.762
Rotation (Longitudinal axis) min speed (°/s)	Cohen's d	26.70585760030	-.536	-.861	-.210
	Hedges' correction	26.84215065910	-.534	-.857	-.209
	Glass's delta	29.60993768489	-.484	-.812	-.153
Rotation (Longitudinal axis) speed harmony (°/s)	Cohen's d	.139164272721	-.156	-.477	.165
	Hedges' correction	.139874496099	-.156	-.474	.164
	Glass's delta	.139813361347	-.156	-.476	.166
Laterization (Sagittal axis) Total Length (°)	Cohen's d	20.96161621741	.988	.648	1.326
	Hedges' correction	21.06859360172	.983	.644	1.320
	Glass's delta	23.93475987780	.866	.514	1.212
Laterization (Sagittal axis) angular velocity (°/s)	Cohen's d	11.945828412595	.706	.375	1.035
	Hedges' correction	12.006793820210	.703	.373	1.030
	Glass's delta	13.692978735897	.616	.279	.949
Laterization (Sagittal axis) max range (°)	Cohen's d	14.36564911018	1.066	.722	1.407
	Hedges' correction	14.43896404686	1.060	.718	1.399
	Glass's delta	16.02268927451	.956	.598	1.308
Laterization (Sagittal axis) max value (°)	Cohen's d	7.49687125402	1.099	.753	1.441
	Hedges' correction	7.53513145633	1.093	.749	1.433
	Glass's delta	8.15858808119	1.009	.648	1.366

Laterization (Sagittal axis) min value (°)	Cohen's d	7.550841961114	-.937	-1.273	-.598
	Hedges' correction	7.589377602353	-.932	-1.266	-.595
	Glass's delta	8.453715272320	-.837	-1.182	-.487
Laterization (Sagittal axis) max speed (°/s)	Cohen's d	22.97898558692	.685	.355	1.014
	Hedges' correction	23.09625859424	.682	.353	1.009
	Glass's delta	25.72353547178	.612	.276	.945
Laterization (Sagittal axis) min speed (°/s)	Cohen's d	22.42517436758	-.688	-1.016	-.357
	Hedges' correction	22.53962100526	-.685	-1.011	-.356
	Glass's delta	24.47786501594	-.630	-.964	-.293
Laterization (Sagittal axis) speed harmony (°/s)	Cohen's d	.147598542716	.145	-.176	.465
	Hedges' correction	.148351810301	.144	-.175	.463
	Glass's delta	.143678263342	.149	-.173	.469

a. The denominator used in estimating the effect sizes.
Cohen's d uses the pooled standard deviation.
Hedges' correction uses the pooled standard deviation, plus a correction factor.
Glass's delta uses the sample standard deviation of the control group.
